# REMINDER program: a randomized controlled trial protocol of a neuropsychological intervention for lifestyle modification in older adults at risk of dementia

**DOI:** 10.1186/s12877-025-06714-x

**Published:** 2025-12-10

**Authors:** Ana Rita Silva, Catarina Baptista, Inês Baldeiras, Maria Salomé Pinho, Margarida Lima, Rosa Marina Afonso

**Affiliations:** 1 Center for Research in Neuropsychology and Cognitive-Behavioral Interventions (CINEICC), Faculty of Psychology and Educational Sciences, University of Coimbra, Colégio Novo Street, Coimbra, 3000-115 Portugal; 2 Center for Innovation in Biomedicine and Biotecnhology (CIBB); Faculty of Medicine, University of Coimbra, Coimbra, Portugal; 3 University of Beira Interior, Covilha, Portugal; 4 Center for Health Technology and Services Research – CINTESIS, Porto, Portugal

**Keywords:** Psychosocial risk factors, Dementia prevention, Older adults, Mild cognitive impairment, Brain health skills

## Abstract

**Background:**

Most dementia risk reduction trials encompass interventions mostly focused in cognitive and health monitoring risk factors, with less focus is given to psychosocial risk factors (e.g. social isolation, depression, anxiety) which can contribute to impoverished engagement in brain protective lifestyles. The REMINDER program was designed as a multimodal intervention that integrates cognitive training, lifestyle psychoeducation, social engagement, emotion regulation, and goal setting, following the framework of internationally established dementia prevention guidelines.

**Aims:**

This manuscript presents the protocol for two randomized controlled trials validating the REMINDER program in both community and clinical populations.

**Methods:**

The REMINDER study includes a Community Trial (cognitively unimpaired at-risk older adults; *N* = 270) and a Clinical Trial (individuals with Mild Cognitive Impairment; *N* = 270). The Clinical Trial includes an additional arm combining caregiver education and support with the REMINDER program. Participants will attend 20 group sessions and complete pre-, post- and follow-up outcome assessments. The program is delivered by trained neuropsychologists to ensure fidelity and high-qualit implementation. Primary outcomes are global cognitive function (composite of MMSE, Word List Total Recall, Category Fluency total, and Trail Making Test Part B) and healthy lifestyle behaviors (HLAT). Secondary outcomes include additional cognitive measures (ACE-R, BRIEF-A, Digit Span), functional status (IAFAI, UPSA-Brief), psychosocial and mental health indicators (GDS-30, WHOQOL-OLD-7, LSNS-6, CORE, MCLHB-DRR), and blood-based markers of neurodegeneration (NfL, GFAP, sPDGFRβ). An exploratory outcome will monitor diagnostic transitions in the MCI subgroup (conversion to dementia) across the 18-month follow-up.

**Conclusions:**

The REMINDER program contributes to dementia prevention research by combining cognitive, lifestyle, and psychosocial strategies within a group-based format, aiming to foster long-term adherence to brain-protective behaviors. Nevertheless, challenges are anticipated, including participant heterogeneity, adherence to lifestyle change, potential drop-out during follow-up, and the complexity of monitoring multiple outcomes. Addressing these limitations will be crucial to inform the future scalability and sustainability of dementia prevention interventions.

**Trial registration:**

ClinicalTrial.gov Identifier NCT05296980, 22/02/2022).

## Introduction

### Background and rationale

People living with dementia and Mild Cognitive Impairment (MCI) may face significant impacts on well-being, the ability of daily living activities and social functioning [1]. The prevalence of dementia is rising, posing a significant public health burden. Globally, affects over 50 million people worldwide [2]. This number is projected to triple by 2050, particularly in low- and middle-income countries, with substantial economic and social costs [3]. In Portugal, around 6% of individuals with 60 or more years old have one type of dementia, and this percentage tends to increase to 9% by 2037, with Alzheimer’s Disease representing 70% of these cases [4, 5]. Dementia prevalence in Portugal is higher than the OECD average, and projections indicate a substantial increase in the coming decades [6], particularly among those aged more than 80 years and women [7]. Current estimates suggest between 85,000 and 218,000 community-dwelling people with dementia, depending on diagnostic criteria [8]. In the meantime, interventions determined to prevent and/or delay the onset of dementia and the elimination, reduced exposure to, and/or a better management of several common modifiable risk factors, have been developed with the purpose to potentially prevent around a third of all dementia cases [9, 10]. In fact, despite the absence of a cure, evidence suggests that 45% of dementia cases are attributable to modifiable risk factors (low education in early life and low cognitive reserve, hearing loss, high LDL cholesterol, traumatic brain injury, hypertension, obesity, excessive alcohol consumption in midlife, diabetes mellitus, depression/stress, physical inactivity, smoking, social isolation, visual loss, and exposure to air pollution in later life), altogether presenting a crucial opportunity for prevention [11]. Within these factors, one can identify some psychosocial risk factors like stress, social isolation, and depression, which are impactful factors for individuals’ mental health [12].

Promising multidimensional interventions, like the FINGER trial – Finish Geriatric Intervention Study to Prevent Cognitive Impairment and Disability, demonstrate the potential to reduce dementia risk [13, 14]. This was the first large multidomain randomized controlled trial (RCT), with 1259 individuals and longitudinal RCT, two years. To date, results demonstrated that a multidomain intervention, including diet, exercise, cognitive training, and monitoring vascular risk, can improve or maintain cognitive functioning in older people from the general population at risk of dementia [14]. Other multidomain interventions have been targeting factors as diet, exercise, cognitive training, and vascular risk [14–16]. However, there are several scientific gaps that hinder a wider and more effective implementation of dementia risk reduction (DRR) programs in large scale and with diverse populations. On one side, drop-out rates in some of the FINGER-like trials exceeded 35%, highlighting the need for approaches to increase engagement and adherence, and the need for disentangling the reasons for low adherence to these programs [17, 18]. On the other side, existing interventions, at least those providing clear information in research papers regarding the program contents and structure, often tend to prioritize physical and cognitive interventions, with relatively less emphasis on psychosocial factors. However, this picture is beginning to change, as recent clinical trials have started to integrate mental health and psychosocial components (e.g [19]).,, acknowledging their significant contribution to dementia risk and their potential impact on engagement with health interventions. Finally, most of the available studies exploring the efficacy of dementia prevention programs focus only on healthy older adults or older adults with a significant number of co-occurring modifiable and non-modifiable risk factors, while those with MCI are at a particularly higher risk of developing dementia and are relatively underrepresented in intervention studies [20]. In fact, this pre-clinical stage of dementia is a clinical group, increasingly conveniently followed in clinical settings with efforts in early diagnosis and monitoring, but there is a lack of focus on preventive interventions for this group at the highest risk of developing dementia (the annual rate of conversion between MCI and Alzheimer’s rounds 10 to 15%) [21]. Recent research demonstrates the value of multidimensional interventions to prevent cognitive deterioration and ameliorate overall functioning and well-being, particularly if it enrolls also the caregivers with psychoeducation approaches [22–24].

Randomized controlled trials (RCT) in the prevention of dementia provide the highest level of evidence to evaluate change after a program aimed to reduce dementia risk, however, it is challenging to conduct such studies in this field, especially due to duration of the interventions and also to population heterogeneity [9, 25, 26]. Nonetheless, so far, the existing interventions have mainly evaluated the effects on global cognition or on specific domains [14–16, 27], as well as in the incidence of dementia, which is an outcome with poor reliability regarding the length of the studies and the time for conversion expected in at-risk populations. However, besides cognition, these interventions should be directed to lifestyle changes to reduce modifiable dementia risk, therefore being necessary to assess whether individuals participating in DRR trials effectively adopt a more protective lifestyle for their brain and global health [28]. However, to our knowledge, most DRR trials have not examined lifestyle and behavior change as part of their main outcomes, which has been stated to be critical to long-term health indicators [29].

In Portugal, despite the high and growing prevalence of dementia, public awareness of modifiable risk factors and preventive strategies remains limited. Barriers such as regional disparities in access to care, socioeconomic inequalities, low health literacy, and limited digital skills may hinder participation in preventive initiatives. These challenges highlight the importance of tailoring brain health programs to local realities while ensuring accessibility, cultural relevance, and engagement. In the face of the current opportunities and challenges brought by the existing dementia prevention trials, most belonging to the World-Wide FINGERS network [30], we developed a group-based 10-week multidomain neuropsychological program – REMINDER – aimed to promoting healthy lifestyles by empowering brain and mental health. The program was informed by principles of holistic neuropsychological rehabilitation [31] and was designed to address barriers identified in the Portuguese context, integrating group-based sessions, clear and accessible language, and minimal reliance on technology to maximize feasibility and inclusiveness across diverse community and clinical settings. The program integrates cognitive training, psychoeducation on lifestyle management strategies (e.g., physical activity, diet, sleep hygiene), emotional regulation techniques, social engagement, and goal setting. Together, these components are expected to improve cognitive performance, promote healthier lifestyle habits, and strengthen emotional well-being and social connectedness through structured group-based activities. These additional components were deliberately integrated to address gaps identified in previous dementia risk reduction trials. Goal setting and monitoring are expected to enhance motivation and adherence to lifestyle changes, a known challenge in multidomain interventions [32, 33]. Emotion regulation strategies directly target psychosocial risk factors such as stress and depression, which are increasingly recognized as contributors to dementia risk [34]. Peer support and group interaction aim to foster social engagement and strengthen cognitive reserve [35, 36], addressing isolation and low stimulation, which have been linked to higher dementia risk [37]. Each weekly session combines psychoeducational content with practical exercises and group discussions, encouraging participants to adopt and maintain brain-health behaviors in daily life. Preliminary findings from a pilot feasibility trial [38] with forty-four community-dwelling at-risk individuals are encouraging. The study achieved a recruitment rate of 69%, a retention rate of 100%, and a high adherence, with participants attending on average 17 out of 20 sessions (85%). Preliminary evidence also suggests improvements in global cognitive performance and in lifestyle behaviors, particularly physical activity engagement, although these findings require confirmation in larger trials. Because the pilot study involved a small, non-randomized sample and was not designed to assess efficacy, further validation is needed. To address this, we are planning a RCT to evaluate REMINDER’s efficacy in a larger sample, including both community-dwelling individuals aged 60 years or older and those diagnosed with MCI, who are at higher risk of progression to dementia. Following recent studies involving brain health programs for MCI patients [23], this planned RCT will test REMINDER’s value across both community and clinical settings, aiming to prevent or delay the onset of dementia.

These limitations in current RCTs underscore the need for innovative and feasible outcome measures in dementia prevention trials. In this regard, recent advances in ultrasensitive techniques, such as the Single Molecule Array (SiMoA), allow accurate measurement of brain-derived proteins in blood samples [39], making it possible to track processes such as neurodegeneration, neuroinflammation, and vascular dysfunction in a minimally invasive way. These biomarkers represent an ideal tool for longitudinal monitoring of brain health and may help overcome current diagnostic and economic barriers [40]. Thus, integrating accessible biomarkers alongside behavioral and cognitive measures may strengthen dementia prevention trials, providing both mechanistic insight and clinically meaningful outcomes.

Given that the REMINDER program will be primarily tested in Portugal, to our knowledge, it will be the first dementia prevention trial to be tested in our country. This will consider the specificities of the Portuguese-aged populations (low levels of education, a significant number of regions with low to very low population density, etc.) enabling the potential transfer of these findings to other comparable cultural/contextual realities. Furthermore, while some international studies have investigated diagnostic transitions in MCI populations (e.g [41, 42]).,, evidence in Portugal remains scarce. This lack of longitudinal data on disease progression highlights the need for studies that monitor diagnostic transitions, which represent a clinically meaningful outcome in individuals at high risk of developing dementia.

### Objectives

Therefore, our aims are:

Primary outcomes.

- To test the efficacy of the REMINDER program in improving global cognition and protective lifestyle behaviors (e.g., physical activity, diet, social engagement, sleep hygiene, cognitive stimulantion, and regular health monitoring) in individuals at risk of dementia (community-dwelling or MCI) at immediate, short- and long-term follow-up.

Secondary outcomes.

- To examine the efficacy of the REMINDER program on neuropsychological functioning, including emotional, cognitive, and functional domains;
- To evaluate the additive effect of including REMINDER4Carers to the REMINDER program on lifestyle behavior, cognition, and well-being of MCI patients;
- To examine the effects of the REMINDER program on markers of neurodegeneration (blood-based biomarkers and neuroimaging markers).

Exploratory outcome.

- To monitor diagnostic transitions in the MCI subgroup (conversion to dementia) across the 18-month follow-up, and examine whether participation in REMINDER alone or and REMINDER with caregiver support may delay progression compared to the waiting list control.

#### Hypotheses

We hypothesize that REMINDER participants will exhibit significant and sustained positive changes in lifestyle behaviors and global cognition, compared to the waitlist control group. For a subset of MCI participants, we will offer an additional condition involving the patient’s partner’s participation in REMINDER. We hypothesize that this complementary support (REMINDER4Carers) will lead to greater improvement in the primary outcome compared to REMINDER alone, according with evidence highlighting the role of care partners in behavior change interventions [22, 24]. We anticipate positive impacts in secondary psychosocial and cognitive aims as well. Additionally, in the MCI subgroup, we hypothesize that participation in REMINDER (with or without care partner involvement) will be associated with a lower rate of diagnostic transition to dementia across the 18-month follow-up.

### Trials design

The study will assess the program’s efficacy in healthy at-risk individuals and those with MCI across community and clinical settings. To adress our research questions, this project employs two superiority randomized controlled trials (RCTs) running in parallel. The Community Trial targets at-risk healthy individuals (*N* = 270) and will be a three-arm, single-blind RCT with 1:1:1 randomization: (1) REMINDER face-to-face, (2) REMINDER virtual, and (3) waiting list control group without active intervention. The Clinical Trial targets individuals with MCI (*N* = 270) and will be a three-arm RCT with 1:1:1 randomization: (1) REMINDER alone, (2) REMINDER with caregiver support, and (3) waitlist control group.

We chose to investigate both community-dwelling at-risk individuals and patients with MCI in order to cover the continuum of cognitive decline prior to dementia, as both groups represent critical targets for prevention but remain underrepresented in multidomain intervention trials.

## Methods

### Participants, interventions, and outcomes

#### Study setting

This project will take place in both rural and urban centres in Portugal. In this region, health services constraints (e.g., lack of primary care doctors for all users, lack of physical settings to provide prevention interventions) and an economic and political crisis have led to underfunding for health prevention proposals. This has disrupted the continuity of support for the current project, despite the increasing adherence of research and clinical partners to the project. We will utilize established relationships with key stakeholders for effective outreach and dissemination: (1) At-risk older adults in community settings (reinforce prior collaborations with established associations of older adults, partner with city councils, and recruit through social media and public awareness campaigns); (2) Clinical (reinforce and formalize partnerships with key healthcare providers in targeted clinical units).

#### Interventions

##### The REMINDER intervention

The REMINDER protocol was developed based on the existing contents of FINGER-like trials (psychoeducation, cognitive training, physical and nutritional advice), culturally adapted cognitive intervention materials [43], and behavior change techniques focused on increasing engagement and decreasing the impact of psychosocial risk factors. In addiction, its design was informed by principles of holistic neuropsychological rehabilitation, a model traditionally applied in populations with brain injury or neurological disease [44, 45]. Despite its clinical origins, this framework emphasizes personal empowerment, engagement, and meaningful goal-directed activities, which are equally relevant in preventive interventions. Holistic rehabilitation is not deficit-oriented but instead builds on personal strengths, aiming to enhance global neuropsychological functioning. Its core components include person-centered care (through personal goal setting and monitoring), integration of cognitive and psychological interventions (e.g., combining memory training with mood regulation strategies), and the embedding of training in meaningful real-world activities. These principles are expected to increase adherence, motivation, and the adoption of protective lifestyle behaviors, holding potential for neuropsychological protection in individuals at risk of dementia [31].

**Fig. 1:**
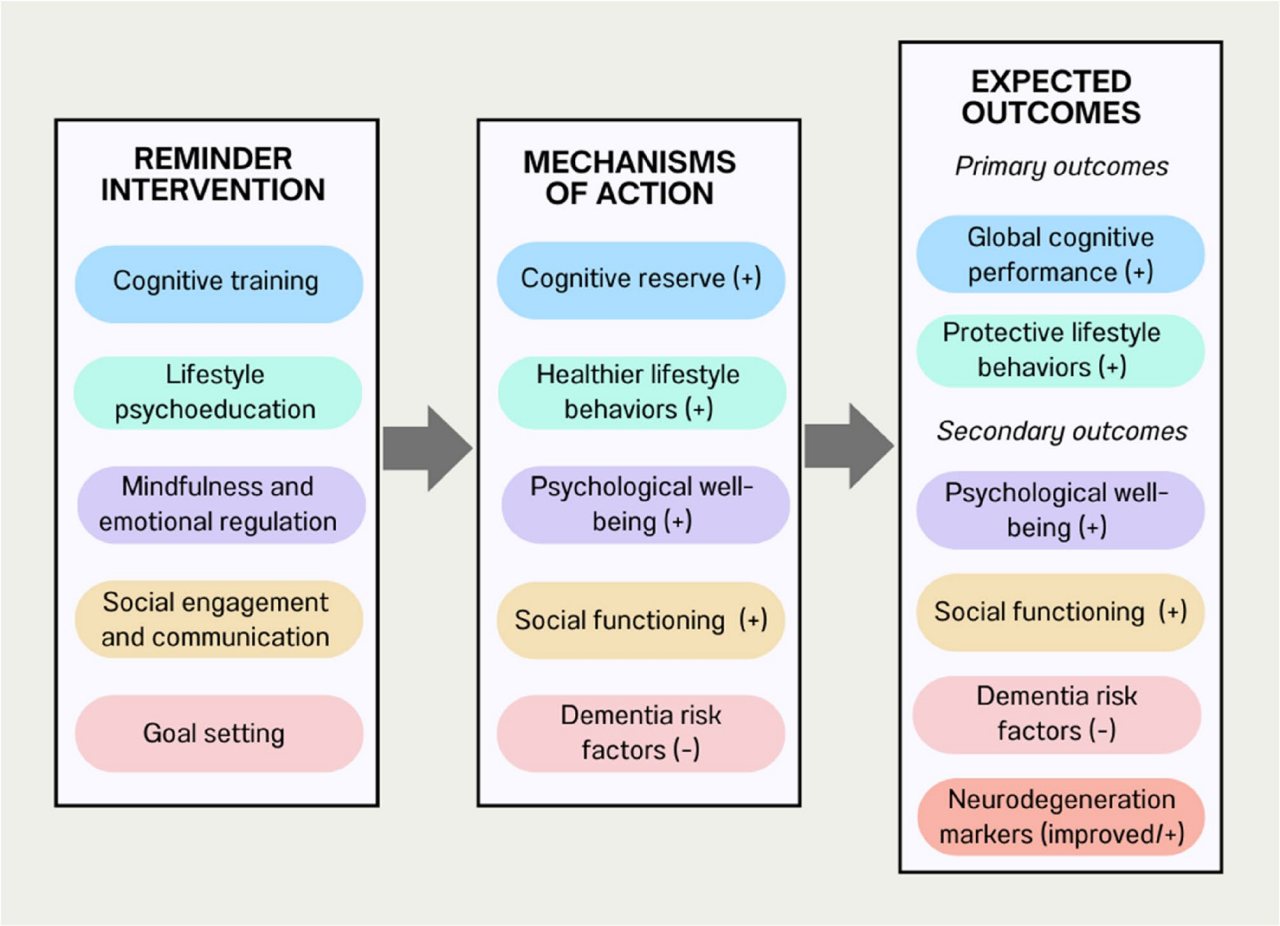
Conceptual model of REMINDER intervention

The REMINDER intervention combines 20 group sessions (with an approximate length of 60–75 min) twice weekly over 10 weeks. To ensure cultural relevance and engagement, sessions integrate Portuguese-specific elements, such as song lyrics from the Festival da Canção, references to the 25th of April revolution, and discussion prompts related to participants’ autobiographical experiences. The content of sessions will integrate brain health psychoeducation, cognitive training, the practice of compensatory memory aids, personally meaningful goals management training, and stress management techniques (see Fig. 1 for the conceptual model of the REMINDER intervention and Table 1 for detailed session contents). Specifically, a set of contents and structures were designed to address existing gaps in the literature. Namely, to overcome low adherence, the program includes interactive and personalized sessions, including strategies like goal setting and mindfulness drawing from previous work with these populations [43, 46], as well as motivational strategies (motivational interviewing, positive reinforcement, and progress tracking) to encourage adherence. In addition, to target specifically the psychosocial risk factors (stress, social isolation, depression, among others) [47], we incorporated stress management techniques based on scripts from compassion-based therapies as well as relaxation techniques [48, 49], social interaction and support and emotion regulation strategies (e.g. attention deployment, positive cognitive reappraisal, reframing speech training). Believing that merging the brain, these behavioral strategies in a dementia risk reduction program will foster individuals’ mental health and build their capacity to draw on improved psychological and cognitive reserve to prevent and cope with remaining risk factors.

A manualized protocol was developed to provide standardized elements and detailed guidelines for program delivery. Health professionals (psychologists, neuropsychologists) delivering the intervention will receive training using the manual before to implementing REMINDER. Postdoctoral-level clinical psychologists from the research team members will lead the interventions in both delivery modes (face-to-face and virtual). To minimize bias, the professionals delivering the program will be counterbalanced across the two delivery methods.

Regarding the sessions’ structure, each one begins with homework revision, followed by a mindful exercise, and ends with a discussion of the homework assignments. All sessions include a psychoeducation task on a specific topic, an experimental exercise, and a moment of sharing and group reflection. During homework revision at the start of each session, participants discuss their experiences with the facilitator, who provides feedback and address any questions. This allows monitoring of adherence and encourages engagement with the intervention.

**Table 1:** Contents of the REMINDER program sessions

Session and Module	Content of the session
Session 1 – Introduction	- Psychoeducation: Presentation of the program and group members; establish group rules; introduction to brain health and mindfulness. - Experimental exercise: First guided mindfulness practice (“Calming Breathing”); Recall the introduction of the participant on the right and their stated characteristic; brief discussion of associative memory principles. - Group reflection: Discussion of expectations and motivations for participation.
Session 2 – Module 1 (REMIND the brain)	- Mindfulness: “Calming Breathing” - Psychoeducation: Overview of modifiable risk and protective factors for cognitive decline. - Experimental exercise: Write and share a “famous character” participants identify with, discuss valued traits, and reflect on self-ideal vs. self-real discrepancies. - Group reflection: Sharing personal experiences with lifestyle habits related to brain health.
Session 3 – Module 1 (REMIND the brain)	- Mindfulness: “Calming Breathing” – soothing - Psychoeducation: Introduction to the brain (structure, hemispheres, lobes, and main functions); Healthy lifestyle recommendations: physical activity, cognitively stimulating activities; diet/nutrition, sleep hygiene, regular health check-ups, and fall prevention. - Experimental exercise: Give authentic, compassionate compliments to the participant on the right for behaviors that protect brain health. - Group reflection: Discussion of barriers and facilitators for maintaining healthy behaviors.
Session 4 – Module 2 (REMIND the goals)	- Mindfulness: “Calming Breathing with Imagination” - Psychoeducation: Importance of personal goals in motivating lifestyle change and long-term commitment. Introduction to SMART goals framework. - Experimental exercise: Each participant defines a personal SMART goal related to brain health and verbally commits to it in front of the group, starting with: “I, (name), aim to …”. This public commitment reinforces shared responsibility and recognition of personal initiative. - Group reflection: Sharing goals with the group; identifying barriers and facilitators to achieving them.
Session 5 – Module 3 (REMIND the attention)	- Mindfulness: “Adapted Body-Scan” - Psychoeducation: Importance of focus and attention for cognitive and emotional well-being, and for achieving personal goals. - Experimental exercise: Practice attentive listening and selective attention tasks to enhance focus. - Group reflection: Discussion of common personal distractors and the importance of attention in daily life.
Session 6 – Module 3 (REMIND the attention)	- Mindfulness: “Body-Centered Mindful Breathing – Body Scan” - Psychoeducation: Recognizing “mind wandering” and automatic pilot behavior; understanding how mindfulness can reduce distractions. - Experimental exercise: Tasks to practice attention control and self-instructions (STOP-FOCUS) to counteract automatic responses. - Group reflection: Identify personal moments of distraction during the week and discuss strategies to maintain focus.
Session 7 – Module 3 (REMIND the attention)	- Mindfulness: “Walking meditation” - Psychoeducation: Deeping understanding of mindful attention, beyond simply being aware or awake; discussing daily benefits of staying focused. - Experimental exercise: Mindful observation of a small food item using all senses to practice present-moment awareness and attention to detail. - Group reflection: Identify and record moments during the week when mindful attention was practiced.
Session 8 – Module 4 (REMIND the memory)	- Mindfulness: “Breathing with imagination” - Psychoeducation: Introduction to memory (it’s functions, its limits, and potential; discussion of common memory problems and protective habits). - Experimental exercise: “Storytelling” task to train memory; group discussion on which types of memory (short-term, semantic, episodic) were exercised. - Group reflection: Brief discussion of participants’ experiences and difficulties with memory preservation.
Session 9 – Module 4 (REMIND the memory)	- Mindfulness: “Breathing with imagination” - Psychoeducation: Introduction to autobiographical memory, life events, reminiscence, and their psychological significance; discussion of how personal interpretations of events affect emotions; benefits of focusing on positive autobiographical memories. - Experimental exercise: Participants share an object, others hypothesize its personal significance, and the owner reveals its meaning. - Group reflection: Discussion of insights from sharing and reflecting on personal memories.
Session 10 – Module 4 (REMIND the memory)	- Mindfulness: “Breathing with imagination and grateful memory” - Psychoeducation: Introduction to reminiscence and life-line mapping; importance of connecting life events to identity, well-being, and positive aging; benefits for family and caregivers. - Experimental exercise: Participants explore autobiographical memory by creating a lifeline of key personal events to reflect on identity and positive aging. - Group reflection: Sharing insights and experiences from the lifeline exercise.
Session 11 – Module 4 (REMIND the memory)	- Mindfulness: “Breathing with imagination” - Psychoeducation: Overview of different memory systems, common forgetfulness, and strategies to address memory challenges. - Experimental exercise: Training in mnemonic strategies for daily life (e.g., visualization, associations, categorization), and discussion of their applicability and difficulties. - Group reflection: Participants share experiences, clarify doubts, and discuss which strategies are most useful or challenging in daily life.
Session 12 – Module 4 (REMIND the memory)	- Mindfulness: “Walking meditation” - Psychoeducation: Introduction to external memory aids (e.g., calendars, notes, reminders) to support planning and recalling future events. - Experimental exercise: Practice using visual mnemonics to remember names and faces through a guided memory exercise with photographs. - Group reflection: Participants share experiences with the exercise, discuss strategies used, difficulties encountered, and perceived benefits.
Session 13 – Module 4 (REMIND the memory)	- Mindfulness: “Mindful walking” - Psychoeducation: Emphasis on external memory aids, specifically agendas/calendars. - Experimental exercise: Classify personal tasks by importance/urgency, record them in a calendar/agenda. - Group reflection: Participants share experiences with the exercise, discuss practical challenges, and reflect on potential application of this method to other compensatory strategies.
Session 14 – Module 5 (REMIND the executive functions)	- Mindfulness: “Mindful walking” - Psychoeducation: Importance of executive functions in daily life and behavioral monitoring; link between executive functions and goal achievement; planning, cognitive flexibility, and problem-solving strategies. - Experimental exercise: Identify which executive functions are required to achieve personal goals; break down goals into smaller, actionable tasks. - Group reflection: Discuss personal difficulties with executive functions and share strategies to overcome them.
Session 15 – Module 5 (REMIND the executive functions)	- Mindfulness: “Progressive Muscle Relaxation” - Psychoeducation: Importance of cognitive flexibility and problem-solving for achieving goals; challenges of goal monitoring and adapting to obstacles. - Experimental exercise: Generate multiple ideas (brainstorm) to solve a specific problem or personal challenge. - Group reflection: Discuss experiences with generating solutions and applying cognitive flexibility in daily life.
Session 16 – Module 5 (REMIND the communication)	- Mindfulness: “Calm Breathing” - Psychoeducation: Importance of communication for brain health and well-being; introduction to common barriers and facilitator strategies. - Experimental exercise: Pair-based communication practice to improve active listening, clear expression, and use of verbal/nonverbal cues. - Group reflection: Sharing personal communication challenges and discussing applications in daily life.
Session 17 – Module 6 (REMIND the socialization)	- Mindfulness: “Breathing together” - Psychoeducation: Role of communication in cooperation and teamwork; importance of socialization for well-being and personal development. - Experimental exercise: Group activities to strengthen cooperation, trust, and positive interactions; sharing three things each participant feels grateful for. - Group reflection: Discussing experiences of social connections and their impact on mood and cognitive health.
Session 18 – Module 7 (REMIND the emotions)	- Mindfulness: “Loving-kindness” breathing - Psychoeducation: Role of emotions in brain health; common emotional regulation difficulties and risk factors associated with aging. - Experimental exercise: Self-portrait activity to recognize personal strengths and limitations, training positive self-concept. - Group reflection: Discuss experiences of emotional challenges and how positive coping strategies support brain and mental health.
Session 19 – Module 7 (REMIND the emotions)	- Mindfulness: “Breathing Love-kindness – compassion” - Psychoeducation: Understanding self-compassion and self-concept during crisis; importance of supportive networks for autonomy and quality of life. - Experimental exercise: Identify personal resilience models; reflect on activities that require support from others and practice self-compassion techniques. - Group reflection: Sharing experiences of resilience and discussing how social support contributes to well-being.
Session 20 – Module 8 (REMIND the learning)	- Mindfulness: Co-create a breathing exercise - Psychoeducation: Review of key cognitive, psychological, and social skills that support brain health and should be practiced daily. - Experimental exercise: Reflecting on personal lifestyle changes and strategies for maintaining them. - Group reflection: Discussing the role of group support in sustaining long-term change and providing a collective evaluation of the program during a shared snack.

##### REMINDER4carers arm

This experimental arm is offered only to MCI participant’s caregivers, who will receive ten parallel psychoeducation and support sessions alongside the delivery of the REMINDER program to the patients. Sessions will be delivered weekly, in groups of 5–8 caregivers, lasting approximately 60 min each, either face-to-face or virtually depending on caregiver’s availability. The sessions will provide emotional support and guidance on caregiving topics such as changing roles, uncertainty of care needs, future and long-term planning, caregiver well-being and self-care, communication skills, and grief and loss.

Research also indicates that increasing caregivers’ awareness of patients’ needs and promoting supportive behaviors enhances patients’ adherence and engagement in protective health behaviors (e.g [35]).,. Moreover, caregivers themselves are at increased risk of cognitive decline and dementia due to chronic stress and caregiving burden [50]. These sessions also aim to promote caregivers’ own well-being and resilience, indirectly supporting their cognitive health.

##### Waiting list control groups

A waitlist control design was selected to ensure provision of the intervention to all participants while allowing comparison with a no-intervention condition over the 6-months follow-up period. After the 6-month follow-up period, participants in the control group will receive the REMINDER intervention, which can be attended either in-person or via videoconferencing, depending on participants’ preference and logistical considerations. Participants’ engagement in other active interventions during the 6-month study period will be monitored through regular follow-up contacts.

#### Outcomes

The professional qualified to conduct pre-and post-intervention neuropsychological assessment will be blind to the intervention arm each participant was assigned. Participants will be assessed at baseline, post-intervention, and at 3, 6, and 18-month follow-up. The endpoint times were selected to capture immediate, short-term, and long-term effects of the REMINDER program on cognitive and lifestyle outcomes, in line with previous multidomain dementia prevention trials.The REMINDER protocol will include a comprehensive protocol of both cognitive, emotional, and functional status (Table 2). The protocol lasts approximately 1-hour session. All assessments will be completed by participants. Care partners will support adherence, provide collateral information when relevant (e.g., diagnostic transitions), and complete the informant version of the BRIEF-A [52]. In addition, they will answer a brief post-intervention satisfaction questionnaire to capture their views on the program’s relevance and utility.

##### Sociodemographic, clinical information

We will collect participants’ sociodemographic (e.g., age, marital status, education level, professional status) and clinical (presence of medical diagnosis, actual medication, sensory issues, mobility deficits, use of substances, and previous issues and hospitalization) information through a questionnaire developed by the researchers. Biological markers of neurodegeneration will also be collected at the baseline and post-intervention.

##### Primary outcome

Given the multidomain nature of this intervention, we will consider as primary outcomes the following:

- A *cognitive function composite *(z-score) will be calculated from the total Mini Mental Status Exam (MMSE) score [53], Word List Total Recall Score (WMS-III) [54], Category Fluency total [55], and Trails Part B [56], providing a measure of global cognitive function.
- *Healthy lifestyle behaviors*, measured with Healthy Lifestyle Assessment Toolkit (HLAT) [57]. This will measure adherence to physical activity, cognitive engagement, and diet and will be complemented with qualitative interviews for a subsample of participants. These outcomes will be measured in all assessment time points (pre-, post-, 3-, 6-, and 18- months follow-up).

These outcomes will be measured in all assessment time points (pre-, post-, 3-, 6-, and 18- months follow-up).

##### Secondary outcomes

The secondary outcome, assessed at baseline, post-intervention, and at 3-, 6-, and 18- months follow-up, will include:

- *Specific cognitive functions* – individual cognitive measures that contribute to the primary cogniticve composite will also be analyzed separately to examine specific domans. These include:
  Global Cognition (Addenbrooke’s Cognitive Examination – Revised [58]); Episodic memory (Word List) [54]; Verbal Initiative (Letters P, M, R and Categories Animals Fluency) [55]; Executive Function (Behavior Rating Inventory of Executive Functions- Adults (BRIEF-A) [52]; Working Memory (Weschler Adult Intelligence Scale (WAIS) – Digit Span Task) [59].
- *Perceived and performance-based functional status*:
  Adults and Older Adults Functional Assessment Inventory (IAFAI) [60]; University of California San Diego Performance-Based Skills Assessment (UPSA Brief) [61].
- *Psychosocial/Mental Health indicators*:
  Geriatric Depression Scale (GDS-30) [62]; World Health Organization Quality of Life (WHOQOL-OLD 7) [63]; Lubben’s Brief Social Network Scale [64]; Psychological change (Clinical Outcome Routine Evaluation) [65]; Motivation (Motivation to Change Lifestyle and Health Behaviour for Dementia Risk Reduction (MCLHB-DRR) [66].
- *Biological markers of neurodegeneration (assessed at baseline, post-intervention): *
  Currently, the most robust blood-based biomarker is the neuronal cytoplasmic protein Neurofilament light chain (NfL). NfL is a sensitive but unspecific marker of axonal injury, with potential ability to rule-in or rule-out neurodegeneration and to assess its progression rate [67]. Therefore, the value of baseline and/or longitudinal measurements of blood NfL in predicting the course of cognitive decline has been verified in different neurodegenerative dementias, and in cognitively normal individuals [68]. Importantly, since blood NfL can detect axonal damage that has occurred in the last 3 months, it has also been used as an indicator of the treatment efficacy of disease-modifying treatments [69]. Other recognized important markers are neuroinflammation and cerebrovascular function alterations and/or blood-brain barrier (BBB) damage. Glial fibrillary acidic protein (GFAP), an intermediate filament protein mainly expressed in astrocytes of the central nervous system and soluble platelet-derived growth factor receptor-β (sPDGFRβ) a marker of BBB-associated capillary mural cell pericyte, respectively, are good candidates [70]. Such markers have also recently shown potential as early surrogates of cognitive dysfunction [71–73]. We will assess the levels of blood NfL, GFAP and sPDGFRβ in a subset of the study population (33% in all study arms) before and after the intervention period. Blood samples will be collected into serum separation tubes, centrifuged at 1500 g, 4^o^C for 10 min. Serum will be separated, aliquoted and frozen at –80^o^C in the same day of collection. Serum NfL and GFAP will be quantified simultaneously using the SiMoA (Single Molecule Array) Neuro-2Plex B multiplex kit in an SR-X platform (Quanterix, USA). Serum sPDGFRβ levels will be determined separately using an already described ELISA kit.
- *Diagnostic transitions in MCI:*
  Conversion from MCI to dementia across the 18-month follow-up, assessed via clinical evaluation and standardized cognitive assessments (e.g., ACE-R, MMSE, clinical interview). This outcome will allow examination of whether participation in REMINDER alone or REMINDER with caregiver support delays progression compared to the waiting list control.

**Table 2:** *Measures*,* psychometric properties*,* and timepoints of assessment*

Construct and measure	Scoring	Criteria/Psychometric properties	Timepoint
Demographic characteristics			
Age, gender, education level, professional status, marital status	—	—	Screening
Clinical characteristics			
Presence of medical diagnosis, actual medication, sensory issues, mobility deficits, use of substances, previous issues and hospitalization, and Lifestyle for Brain Health (LIBRA) score [51]	LIBRA score: range from − 5.9 to 12.7	LIBRA score: higher = more risk	Screening
Primary Outcome			
* Cognitive function composite*			
Mini-mental State Examination (MMSE)	Total 0–30	Higher scores indicating better functioning	Baseline, post, 3, 6, and 18-month follow-up
Word List (WMS-III)	Sum of recalled words	Internal consistency 0.67 to 0.90 (median = 0.83)	Baseline, post, 3, 6, and 18-month follow-up
Verbal Fluency (letters and animals)	Number of correct words in 60s	Excellent reliability: α = 0.89; ICC = 0.996	Baseline, post, 3, 6, and 18-month follow-up
Trail Part B	Time to completion (sec)	Higher times = worse performance	Baseline, post, 3, 6, and 18-month follow-up
* Health lifestyle behaviors*			
Healthy Lifestyle Assessment Toolkit (HLAT)	Traffic light rating system for 8 lifestyle components: low risk (green), moderate risk (yellow), relevant risk (orange)	—	Baseline, post, 3, 6, and 18-month follow-up
Secondary outcome			
* Specific cognitive functions*:			
Addenbrooke’s Cognitive Examination – Revised (ACE-R)	Total 0–100 (with 5 subdomains)	Higher scores indicating better functioning; Excellent internal consistency (Cronbach’s α = 0.95)	Baseline, post, 3, 6, and 18-month follow-up
Behavior Rating Inventory of Executive Functions - Adults (BRIEF-A)	3-point Likert scale (1 = Never; 2 = Sometimes; 3 = Often), frequency of behaviors in the past month	Indexes and Global Executive Composite: α = ≥0.90 (very good); Scales: α = 0.70–0.80.70.80 (acceptable) or α = ≥0.80 (good)	Baseline, post, 3, 6, and 18-month follow-up
Digit Span Task - Weschler Adult Intelligence Scale (WAIS)	Total correct sequences: Forward (max = 16); Backward (max = 14); Digit memory total (max = 30)	—	Baseline, post, 3, 6, and 18-month follow-up
*Perceived and performance-based functional status*:			
Adults and Older Adults Functional Assessment Inventory (IAFAI)	50 items (0 = no functional limitation, 1 = functional limitation)	Global and domain-specific functional limitations (physical, cognitive, emotional)	Baseline, post, 3, 6, and 18-month follow-up
University of California San Diego Performance-Based Skills Assessment (UPSA Brief)	Performance score 0–100	Adequate internal consistency α = 0.86	Baseline, post, 3, 6, and 18-month follow-up
* Psychosocial/Mental health indicators*:			
Geriatric Depression Scale (GDS-30)	Sum of 30 dichotomous items (0–30)	Excellent reliability: α = 0.91; inter-rater Kappa = 0.87; good validity	Baseline, post, 3, 6, and 18-month follow-up
World Health Organization Quality of Life (WHOQOL-OLD 7)	Domain scores 8–40	Higher = better quality of life	Baseline, post, 3, 6, and 18-month follow-up
Lubben’s Brief Social Network Scale	Total scores range from 0 to 30, with higher scores indicating stronger social networks.	Higher = stronger social networks. Adequate internal consistency (Cronbach’s α = 0.80).	Baseline, post, 3, 6, and 18-month follow-up
Psychological change (Clinical Outcome Routine Evaluation)	0–4 Likert scale per item (0 = Never, 4 = Almost always), reflecting experiences in the past week	Excellent internal consistency: α = 0.94	Baseline, post, 3, 6, and 18-month follow-up
Motivation (Motivation to Change Lifestyle and Health Behaviour for Dementia Risk Reduction (MCLHB-DRR)	27 items on 7 subscales (Health Belief Model)	Moderate-to-high internal reliability: α = 0.608–0.864; test-retest reliability: α = 0.552–0.776	Baseline, post, 3, 6, and 18-month follow-up
* Biological markers of neurodegeneration*			
NfL	Pg/mL (normal expected range within 5.0–50.0 pg/ml in older adults)	Higher levels indicate increased axonal loss	Baseline, post-intervention
GFAP	Pg/mL (normal expected range within 50–300 pg/ml in older adults)	Higer leves indicate increased neuroinflammation	Baseline, post-intervention
sPDGFRβ	Pg/mL	Higher levels indicate impaired blood-brain barrier funcion	Baseline, post-intervention
Exploratory outcome (MCI subgroup)			
* Diagnostic transitions*	Conversion from MCI to dementia	Assessed via clinical evaluation, standardized cognitive assessments (ACE-R, MMSE, clinical interview).	Baseline, post, 3, 6, and 18-month follow-up

#### Participants

**Fig. 2:**
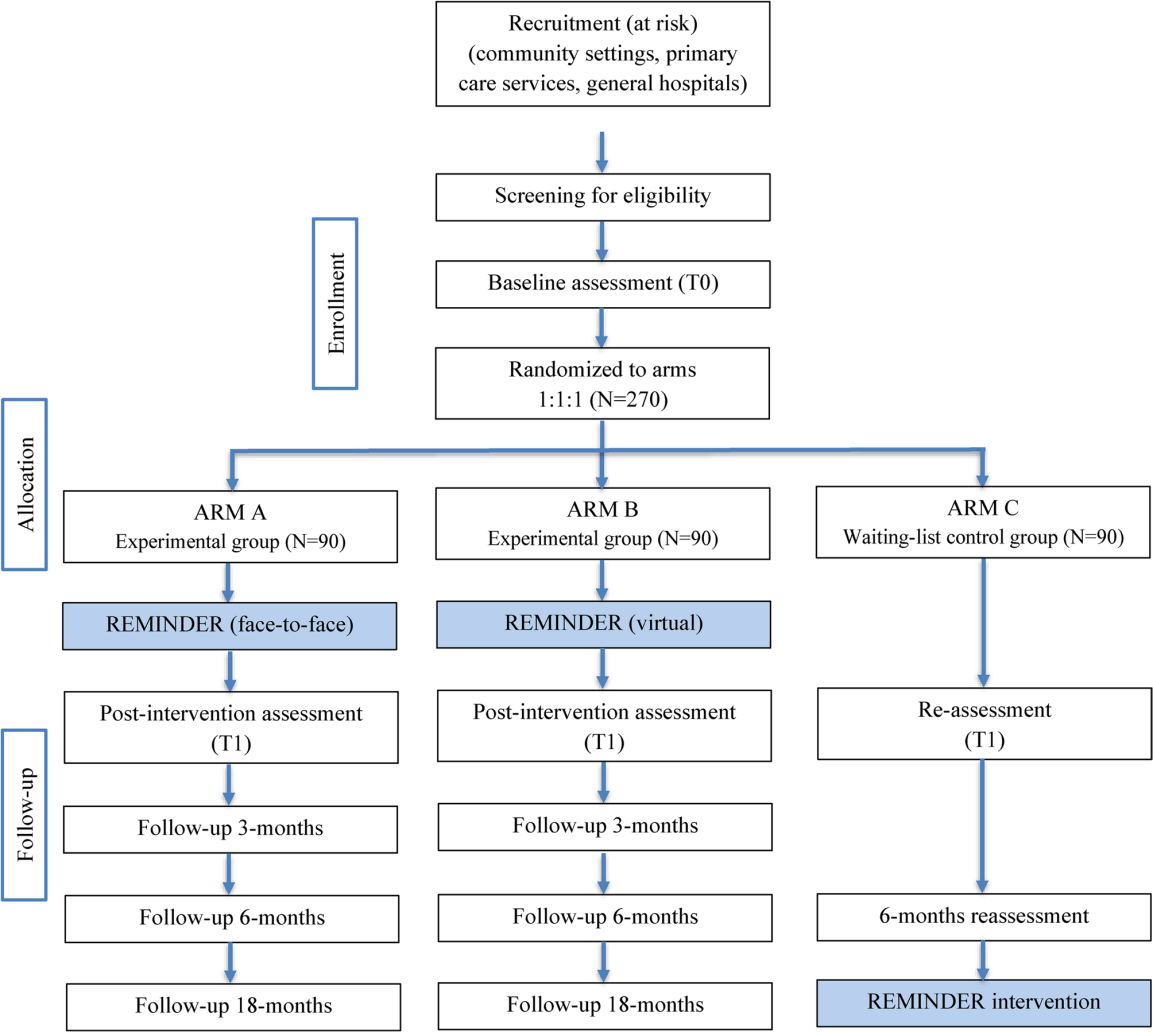
Flowchart of participants in the Community Trial

**Fig. 3:**
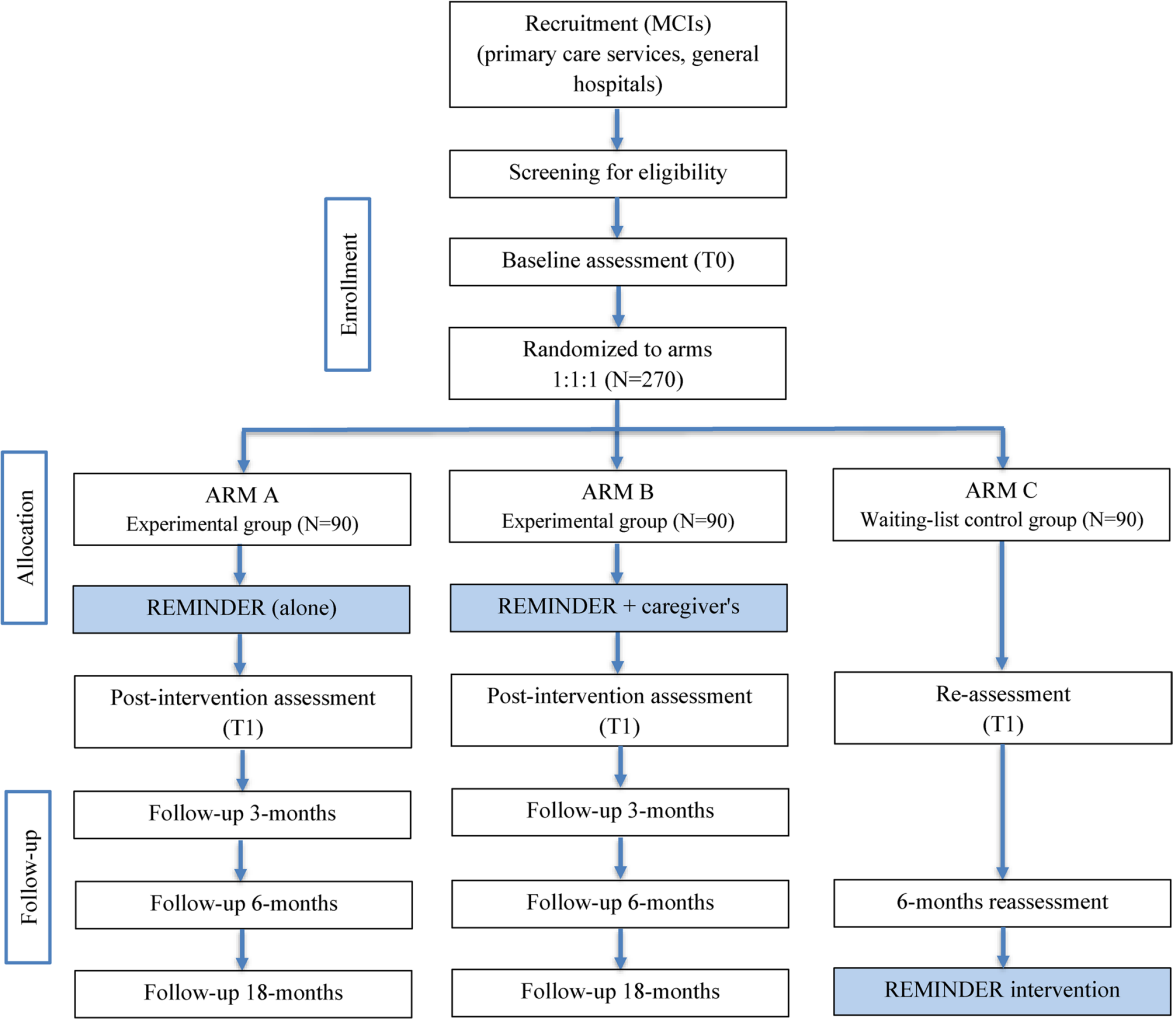
Flowchart of participants in the MCI trial

This study follows a randomized, waitlist-controlled design. Eligible participants will be invited to complete the baseline assessment. Recruitment will target individuals presenting modifiable dementia risk factors, as well as caregivers/relatives of MCI patients, who will be included in a dedicated experimental arm of the Clinical Trial. Following baseline assessment, participants will be randomly assigned to one of the study arms: *Community Trial –* REMINDER face-to-face, REMINDER virtual, or Waitlist Control (Fig. 2, trial flowchart); *Clinical Trial –* REMINDER intervention alone, REMINDER plus caregiver support, or Waitlist Control (Fig. 3, trial flowchart). A researcher blind to the assessment procedure will conduct randomization, and then all participants will be informed about their assigned condition. Waitlist participants will be offered the REMINDER intervention after 6 months, while all participants will be followed for 18 months post-intervention (24 months total).

#### Eligibility criteria

Participants or their representatives must give written, informed consent before initiating any study procedures (see Appendix 1 for Informed Consent Form).

##### Inclusion criteria

For all participants:

1. Age 60–80 years, to focus on individuals in the preclinical or early-risk stage for cognitive decline;
2. Dementia Risk Score ≥ + 2 (LIBRA index) [74], to include participants with elevated dementia risk, in line with the preventive aim of the program;
3. Elementary reading and writing skills, to ensure comprehension of intervention materials and participation in assessments;
4. Basic digital literacy or access to a caregiver supporting videoconferencing, to enable participation in remote sessions and online components.

Community (cognitively intact) sample clarification: Participants recruited for the community arm are cognitively intact at baseline and do not meet MCI criteria. This ensures that the community sample represents preclinical or at-risk individuals without a formal diagnosis of MCI, clearly distinguishing them from the clinical trial group.

Additional criteria for participants with MCI (clinical arm):

1. Meeting one of the adapted CERAD criteria for very mild cognitive impairment [i) MMSE between 21 and 28 points, ii) memory learning task (3 × 16 words) of 25 words or less, iii) delayed recall: 75% or less];
2. Clinical diagnosis of MCI as defined by the National Institute on Aging-Alzheimer’s Association workgroups - NIA-AA [75];
3. Availability of a caregiver (living with or spending at least 10 h per week with the participant) to support participation and adherence. Participants without an available caregiver will still be eligible to receive the REMINDER program after the conclusion of the RCT.

###### Exclusion criteria

Exclusion criteria for both trials will include:

1. History of neurological or psychiatric conditions likely to affect cognition (e.g., stroke, major depression);
2. Sensory deficits or mobility limitations preventing the effective delivery of the assessment or intervention;
3. Significant functional deficits compromising engagement in program activities;
4. Diagnosis of dementia, as the program is preventive.

#### Sample size

The sample size estimation was performed in “G*Power”, a statistical software program. Power analysis was performed and was based on both existing data on the effects of dementia risk reduction programs in a cognitive composite [28, 76] as well as in our preliminary feasibility study results that held small to medium effects (f >0.20). Accordingly, for the Community trial, a total sample size of 244 was determined based on an expected power of 0.80 to detect small to medium effects (f >0.20) in comparison analyses in the primary outcomes (e.g., cognitive composite; lifestyle change) (G*Power). Considering an expected dropout rate of 10%, a total sample size of 268 participants leads to an approximate required final expected sample size of 270 (90 per arm). For the Clinical Trial, the same power analysis was performed, and considering now three arms, a final sample size of 270 is distributed across arms, with 90 participants per arm.

Given the multifactorial higher risk of women developing dementia, we will take this into account in the sex/gender ratio of the sample recruited as well as in the efficacy analyses.

#### Recruitment

For the Community trial, potential participants will be contacted through traditional and social network advertisement (Project’s website - www.remindereomeucerebro), as well as through community and clinical recruitment settings with established protocols with the University of Coimbra (Coimbra University Hospitals, Memory Clinics, Elderly Day Care Centres, and Senior Universities in the Coimbra region). Interested individuals will first be contacted by a trained member of the research team, either by phone or in person, to provide detailed study information and conduct an initial eligibility screening. Those who appear eligible will be invited to an in-person meeting, where full eligibility will be confirmed, written informed consent will be obtained, and participants will complete the baseline assessment before random assignment to study arms.

For the Clinical trial, general hospitals from three Portuguese regions (Coimbra, Trás-os-Montes, Porto) will be approached (following approval from each Local Health Unit’s ethics committee). Doctors will identify and signal patients with a diagnosis of MCI. These patients will then be approached by a member of the research team, who will explain the study, conduct the screening (during a clinic visit), and schedule the baseline assessment for those eligible. After signing written informed consent, participants will complete the baseline evaluation and will then be randomly allocated to the study arms.

In both trials, study ID numbers will be assigned by a research team member using a pre-generated list of random numbers. These IDs are used solely for anonymization and data management purposes, and are unrelated to the randomization of participants into study arms.

Recruitment is anticipated to begin in January 2026, with data collection (including pre- and post-intervention assessments and follow-up assessments at 3, 6, and 18 months) expected to continue until approximately mid-2027.

### Assignment of interventions

Once participants meet all inclusion criteria and provide informed consent, they are assigned a unique study ID number. For randomization, a predetermined set of study ID numbers is allocated to each recruitment site based on the expected recruitment ratio. Randomization is then performed by a researcher blinded to assessment procedures using a computerized random number generator, assigning participants to their respective study groups: Community Trial - REMINDER face-to-face, REMINDER virtual, or Waitlist Control and Clinical Trial - REMINDER alone, REMINDER + caregiver support, or Waitlist Control. This process ensures allocation concealment and prevents bias in group allocation.

#### Implementation

Recruitment staff request the randomization from the central study team and receive a form with the assigned randomization number, which is forwarded to another team member responsible for informing the participant. Participants are then notified of their assigned group. Throughout this process, recruitment staff and outcome assessors remain blinded to the allocation, maintaining the integrity of the trial.

#### Blinding (masking)

A blinded trained neuropsychologist will perform the neuropsychological assessment at the predicted time points, and another trained clinical neuropsychologist will deliver the REMINDER program under the supervision of the PI and remaining research team, distributed across recruitment sites. This researcher and the study coordinator will be responsible for database creation and completion, and the remaining research team will perform the data analysis.

#### Statistical methods

Statistically, we will analyse the program efficacy following intention-to-treat (ITT) and per-protocol (PP) principles following CONSORT recommendations [77], allowing to examine data from all randomized participants for the two trials (even those with missing values on outcome measures). Linear mixed models (LMMs) will be conducted to determine the effects of the intervention over time (time × group interaction effects) on primary and secondary outcomes. LMMs have been chosen as the primary method for analyzing repeated measures data, as they account for within-subject correlations over time, allow inclusion of fixed and random effects, and handle missing data under the missing-at-random assumption. Given that our primary and secondary outcomes are continuous variables, LMMs are appropriate. Should exploratory analyses indicate non-normality or different data distributions, alternative mixed models (e.g., generalized linear mixed models) will be considered to ensure model adequacy. Participant characteristics, including age, sex/gender, education level, baseline cognitive performance, baseline dementia risk, and adherence to the intervention (attendance and engagement in sessions), will be included as covariates to account for potential confounding effects on outcomes. Additional statistical analyses such as two-wave latent change score models, reliable change index, Chi-square tests, and within-group effect sizes will be performed, as well as a mediator and moderator analysis. The generalized estimating equation (GEE) method will be used to analyse the estimated regression coefficients (i.e., the effect estimates) for these outcomes (functionality, global cognition, dementia risk score) concerning neurobiological data (neural plasticity and NfL). The GEE method extends standard regression analysis, accounts for the correlation between repeated measurements, and is more robust to violations of normality. For exploratory analyses of diagnostic transitions (e.g., from cognitively intact to MCI or dementia), Markov models will be employed to estimate transition probabilities and characterize patterns of change across follow-up. Initial data analysis will be done using IBM SPSS Software, version 28.0 (IBM Corp, Armonk, NY, USA), and more advanced modeling will be performed using R Program (R Core Team, 2022).

## Discussion

Dementia prevention trials may benefit from prioritizing healthy lifestyle interventions, given emerging evidence of their potential impact. The REMINDER program integrates evidence-based strategies from prior multidomain trials, including goal setting, lifestyle psychoeducation, cognitive training, emotion regulation, and social engagement, and adapts them within a holistic neuropsychological rehabilitation framework. Previous feasibility testing of REMINDER in a small community sample demonstrated high adherence and preliminary benefits on cognition and lifestyle behaviors, suggesting that the program is acceptable and feasible for older adults at risk of dementia.

A distinctive feature of REMINDER is it emphasis on psychosocial risk factors, such as depression, stress, and social isolation, which remain underrepresented in most dementia risk reductions trials. By combining cognitive and emotional strategies with opportunities for peer interation and support, the program is designed to foster engagement, sustain motivation, and encourage the adoption of healthier lifestyle bahviors. Goal setting and monitoring, in particular, may help participants translate program content into everday prative, supporting adherence across time.

A recent clinical trial by Mace et al. (2024), has further highlighted the importance of addressing psychosocial and mental health risk factors in dementia prevention, signaling an important shift in the field. Notably, the REMINDER program was conceived and developed in 2022, prior to the publication of such evidence, and thus reflect an early commitment to integrating these components into a multidomain framework. While both approaches recognize the relevance of psychosocial risk factors, REMINDER is distinctive in its explicit grounding in holistic neuropsychological rehabilitation principles and its cultural adaptation to the Portuguese context, with an emphasis on goal setting, peer support, and the integration of cognitive and emotional strategies. In this sense, the emerging literature reinforces rather than diminishes the novelty and value of our approach.

Despite this promise, several potential challenges should be considered. Participant adherence may vary depending on individual motivation, baseline cognitive status, and availability of caregiver support. The collection of blood biomarkers may be limited by logistical or acceptability constraints. Additionally, cultural and socioeconomic factors specific to the Portuguese context may limit the generalizability of findings.

The planned RCT will expand upon prior feasibility work by including both community-dwelling older adults and individuals with MCI, allowing examination of program applicability across different risk profiles. In addition to cognitive and psychosocial outcomes, the study will also explore biomarker changes, providing insight into potential mechanisms of action. Together, these findings will contribute to the broader literature on dementia prevention, particularly in culturally and demographically underrepresented populations, and may inform the design of future holistic, multidomain prevention programs.

### Ethics and dissemination

#### Research ethics approval

The Ethics Committee of the Faculty of Psychology and Educational Sciences of the University of Coimbra (CEDI/FPCEUC:62/8) approved this study, registered in Clinical Trials (ClinicalTrial.gov Identifier: NCT05296980, 22/02/2022). The project will be also submitted for approval to the collaborative hospitals Ethics Committees (CHUC – Centro Hospitalar e Universitário de Coimbra, CHTMAD – Centro Hospital Trás os Montes e Alto Douro; ARS-C - Associação Regional de Saúde do Centro; ACES – Grande Porto II – Agrupamento de Centros de Saúde do Porto II) in which the clinical and at-risk sample recruitment will take part.

#### Consent or assent

All participants must sign the approved informed consent forms before any study-related processes.

#### Confidentiality

All participants will be informed about the study-related issues and the confidentiality of the researcher’s duties. However, they must be fully aware of the voluntary nature of their participation and the right to refuse to participate or withdraw at any time and without penalty.

#### Declaration of interest

No potential conflict of interest was reported by the author(s).

#### Access to data

This project will implement data anonymization procedures to protect personal data. Only a limited and well-identified group of people will have access to parts of the data, complying with the General Data Protection Regulation (GDPR) laws. 

## Data Availability

No datasets were generated or analysed during the current study.
